# Epidemiologic analysis of antimicrobial resistance in hospital departments in China from 2022 to 2023

**DOI:** 10.1186/s41043-024-00526-2

**Published:** 2024-03-06

**Authors:** Hui-Jun Bai, Qing-Feng Geng, Fang Jin, Yong-Li Yang

**Affiliations:** 1 Department of Clinical Pharmacy, The Seventh People’s Hospital of Hebei Province, 389 Jungong Road, Xicheng District, Dingzhou, 073000 China; 2 Department of Hospital Office, The Seventh People’s Hospital of Hebei Province, 389 Jungong Road, Xicheng District, Dingzhou, 073000 China; 3 Department of Clinical Laboratory, The Seventh People’s Hospital of Hebei Province, 389 Jungong Road, Xicheng District, Dingzhou, 073000 China

**Keywords:** Multidrug-resistant bacteria, Antimicrobial susceptibility testing, Bacterial drug resistance surveillance, Hospital infections, Penicillin

## Abstract

Bacterial drug resistance monitoring in hospitals is a crucial aspect of healthcare management and a growing concern worldwide. In this study, we analysed the bacterial drug resistance surveillance in our hospital from 2022 Q1 to 2023 Q2. The main sampling sources were respiratory, blood, and urine-based, and the main clinical infections were respiratory and genitourinary in nature. Specimens were inoculated and cultured; bacterial strains were isolated using a VITEK® 2 Compact 60-card automatic microorganism identifier (bioMerieux, Paris, France) and their matching identification cards were identified, and manual tests were supplemented for strain identification. The most common Gram-positive bacteria detected were *Staphylococcus aureus*, followed by *Enterococcus faecalis* (*E*. *faecalis*), *Staphylococcus epidermidis* (*S*. *epidermidis*), and *Staphylococcus haemolyticus* (*S*. *haemolyticus*). The most common Gram-negative bacteria detected were *Escherichia coli*, *Klebsiella pneumoniae*, and *Pseudomonas aeruginosa*. The most prevalent multidrug-resistant bacteria were those producing extended-spectrum beta-lactamases, followed by methicillin-resistant *Staphylococcus aureus*, followed by carbapenem-resistant *Enterobacterales*. This study suggests that the prevention and control of infections in the respiratory and genitourinary systems should be the focus of anti-infective work and that the use of antimicrobials should be reduced and regulated to prevent the emergence and spread of resistant bacteria.

## Introduction

Bacterial drug resistance monitoring in hospitals is a crucial aspect of healthcare management. The World Health Organization (WHO) has reported high levels of resistance in bacteria causing life-threatening bloodstream infections and increasing resistance to treatment involving several bacteria that cause common infections in the community [1]. The Global Antimicrobial Resistance and Use Surveillance System report provides analyses for antimicrobial resistance (AMR) rates in the context of national testing coverage, AMR trends since 2017, and data on antimicrobial consumption in humans in 27 countries. The report shows high levels (above 50%) of resistance were reported concerning bacteria that frequently cause bloodstream infections in hospitals, such as *Klebsiella pneumoniae* (*K*. *pneumoniae*) and *Acinetobacter spp*. These life-threatening infections require treatment with last-resort antibiotics such as carbapenems [2]. The Center for Disease Control and Prevention (CDC) also provides information on AMR and antibiotic use through its National Healthcare Safety Network (NHSN), which is the China’s most widely used healthcare-associated infection tracking system [3]. It provides data to identify problem areas, measures the progress of prevention efforts, and, ultimately, aims to eliminate healthcare-associated infections [4]. This study may provide more clinically relevant resistance information to the CDC and NHSN.

Antibiotic resistance is a growing concern in healthcare management. It occurs when bacteria, viruses, fungi, and parasites change over time and no longer respond to medicines, making infections more difficult to treat and increasing the risk of disease spread, severe illness, and death [5–7]. The consequences of bacterial resistance are serious and can lead to ineffective treatment regimens for common bacterial infections, thereby delaying the treatment of patients, giving rise to complications, and even death [8]. Several types of antibiotic-resistant bacteria have been identified [9, 10]. Methicillin-resistant *Staphylococcus aureus* (MRSA) is resistant to many antibiotics and can cause skin-related and other severe infections [11]. Vancomycin-resistant *Enterococcus* (VRE) is an enterococcus bacteria that is resistant to the antibiotic vancomycin and can cause infections in the urinary tract, bloodstream, and wounds [12]. Multidrug-resistant (MDR) *Pseudomonas aeruginosa* (*P*. *aeruginosa*) is a bacteria type that is resistant to several antibiotics and can cause infections in the lungs, urinary tract, and bloodstream [13]. Carbapenem-resistant *Enterobacterales* (CRE) is a family of bacteria that is resistant to carbapenem antibiotics and can cause infections in the urinary tract, bloodstream, and other parts of the body [14]. The detection rate of MDR bacteria is increasing, and even the emergence of pan-drug-resistant bacteria and fully drug-resistant bacteria. Increasing detection rates of MDR not only poses a threat to the safety of patients but also creates a huge economic burden [15]. Therefore, drug resistance monitoring of clinical isolates cannot only help us to understand the degree of bacterial evolution but also provide an effective and timely basis for the empirical anti-infective treatment and effective control of hospital infections. In this study, we retrospectively analyse the drug resistance data of isolates from clinical patients to provide an effective and timely basis for clinicians with which to empirically deliver anti-infective treatment and effectively control nosocomial infections.

## Methods and materials

### Source of bacterial strains

The current research was retrospectively conducted. The collection and isolation of bacteria were clinically conducted from 1 January 2022 to 30 June 2023 by sending microbiological cultures from various clinical departments of our hospital.

### Instruments and reagents

A Deere DL-96 microbial automatic identification instrument and its matching identification cards (Zhuhai Deere Bioengineering Co., Ltd, China) were used, and a culture medium was purchased from Zhengzhou Antu Bioengineering Co., Ltd. Drug-sensitive paper was purchased from Wenzhou Kangtai Biotechnology Co., Ltd. Drug sensitivity tests were performed and evaluated according to the disk diffusion method or automated instrument method recommended by the Clinical and Laboratory Standards Institute (CLSI) 2023 edition [16].

### Culture and identification methods

Bacterial culture, identification and antimicrobial susceptibility testing were performed following the requirements of the National Clinical Laboratory Practice (3rd edition) [17] for specimen inoculation, bacterial culture, the isolation of bacterial strains by the VITEK® 2 Compact 60-card fully automated microbial identifier and its supporting identification cards, as well as complementary manual tests, such as a 42 °C growth test, oxidase test, catalase test, and a plasma coagulase test. The test methods were performed following the requirements of the National Clinical Laboratory Practice (3rd edition) [17]. The MDR, ESBL, VRE, MRSA was determined by the National Clinical Laboratory Practice (3rd edition) [17]. The Vitek 2 Compact 60-card fully automated microbial identifier can automatically identify more than 400 strains of bacteria including Gram-negative bacilli, Gram-positive bacteria, yeasts, aerobic bacilli, and anaerobic bacteria. Thirty cartridges can be run simultaneously, and susceptibility testing can be performed using susceptibility cards. An antimicrobial drug susceptibility test was performed using the paper diffusion method, and the interpretation standard followed the 2023’s American CLSI M100 document.

### Statistical analysis

The distribution of all the identified bacterial strains was counted according to the genus level, and the percentage of each genus was calculated. All data were statistically analysed using the WHO Bacterial Resistance Surveillance Network software, WHONET 5.4 (repeated isolation of the same pathogenic bacteria from the same site in the same patient was excluded).

## Results

### Detection of multidrug-resistant bacteria

**Fig. 1:**
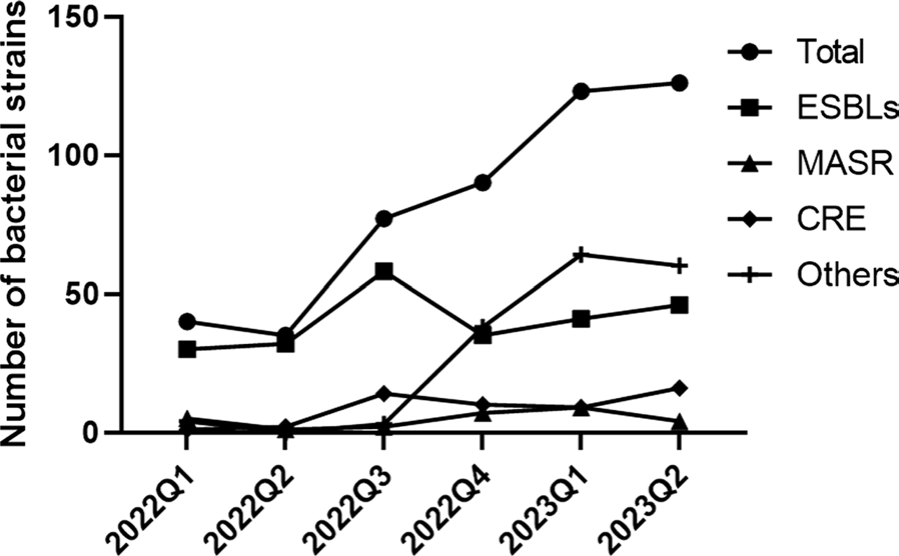
Trend in the number of multi-drug resistant bacteria detected from 2022Q1 to 2023Q2

Between 2022 Q1 and 2023 Q2, the total number of multi-drug resistant bacteria detected in the samples sent for testing by various departments of the hospital showed an increasing trend (Fig. 1) from 40 strains in 2022 Q1 to 126 strains in 2023 Q2, suggesting the need for intervention in antimicrobial drug use in various departments. Among the MDR bacteria detected, the largest number of positive strains were those producing extended-spectrum beta-lactamases (ESBLs), followed by MRSA, followed by CRE with varying degrees of detection, as well as other multi-resistant bacteria. The number of detections of ESBLs, MRSA, and CRE was essentially stable during the monitoring period; however, other multi-resistant bacteria showed a sudden increasing trend, suggesting the need for further analysis of other strains as shown in Fig. 1.

### Sources of specimens of multi-drug resistant bacteria

**Fig. 2:**
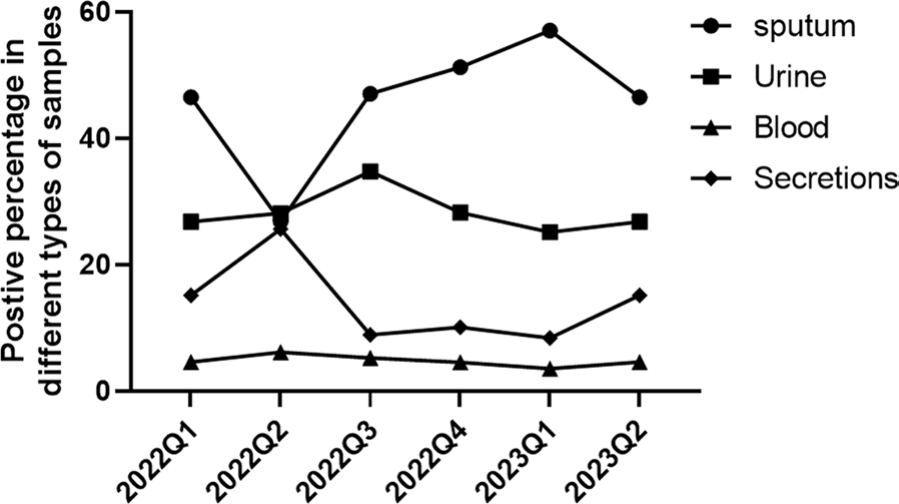
Trend in the number of multi-drug resistant bacteria detected in four types of samples from 2022Q1 to 2023Q2

From 2022 Q1 to 2023 Q2, among the samples sent for testing by various departments in our hospital, the sample type with the highest number of multi-resistant bacteria detected was sputum samples, suggesting that there is a risk of respiratory infections with multi-resistant bacteria in our hospital. The proportion of positive results in sputum samples has remained at a stable high level, suggesting the need for focusing on airborne multi-resistant bacteria. The remaining three sample types had a stable proportion of positive MDR bacteria (Fig. 2). The lowest number of positive MDR bacteria was detected in blood samples. Overall, multi-resistant bacteria in nosocomial patient infections were generally stable, and further control of antimicrobials used by all departments is needed to reduce the emergence of resistant bacteria.

### Specific identification of detected bacterial genera

Details of the various bacteria genera detected in all of the samples sent for analysis during the period from 2022 Q1 to 2023 Q2 are shown in Table 1. The largest proportion of detections were Gram-negative bacteria, representing a total of 1633, accounting for 83.74% of all the bacterial genera detected, indicating the need for a stronger focus on this genus.

**Table 1:** The details of the detected bacteria during 2022Q1 to 2023Q2

Pathogen	2022Q1		2022Q2		2022Q3		2022Q4		2023Q1		2023Q2	
	Number of strains (*n* = 246)	Component ratio	Number of strains (*n* = 291)	Component ratio	Number of strains (*n* = 385)	Component ratio	Number of strains (*n* = 288)	Component ratio	Number of strains (*n* = 370)	Component ratio	Number of strains (*n* = 370)	Component ratio
Gram-positive bacteria	41	16.67	42	14.43	39	10.13	28	9.72	33	8.92	31	8.38
*Staphylococcus aureus*	22		17		15		20		26		22	
*Enterococcus faecalis*	7		13		4		2		5		5	
*Staphylococcus hemolyticus*	4		4		3		0		0		0	
Others	8		8		17		6		2		4	
Gram-negative bacteria	231	93.90	183	62.89	337	87.53	253	87.85	317	85.68	312	84.32
*Klebsiella pneumoniae*	68		37		85		71		101		107	
*Escherichia coli*	63		48		92		75		91		102	
*Pseudomonas aeruginosa*	50		31		64		43		55		45	
*Acinetobacter baumannii*	7		3		10		13		22		23	
Calcium acetate	6		6		13		0		0		3	
*Klebsiella acidophilus*	6		8		4		4		5		0	
*Serratia marcescens*	5		2		2		0		6		0	
*Proteus mirabilis*	5		5		15		9		9		5	
*Enterobacter aerogenes*	4		9		6		0		2		0	
*Serratia marcescens*	4		3		0		0		4		3	
*Stenotrophomonas maltophilia*	3		2		9		11		7		9	
*Enterobacter cloacae*	2		5		16		4		6		11	
*Serratia marcescens*	2		0		0		0		0		0	
*Enterobacter aerogenes*	2		2		0		0		0		0	
*Flavobacterium citrobacter*	2		3		10		4		0		0	
Other bacteria	2		19		11		19		9		4	
Fungi	19	7.72	21	7.22	7	1.82	9	3.13	15	4.05	10	2.70
*Pseudomonas albicans*	12		15		4		5		14		6	
*Pseudomonas tropicalis*	3		3		2		2		1		1	
*Smooth pseudomallei*	4		3		1		2		0		3	

### Major drug-resistant bacteria and their resistance rates to major antimicrobial drugs

#### *Staphylococcus aureus* resistance rate

Bacterial strains and their matching identification cards were identified as being *Staphylococcus aureus* (*S. aureus*) by catalase test, Gram-staining, and a plasma coagulase test, followed by testing using the Vitek 2 automatic microorganism identifier. Among the detected *S. aureus*, the bacteria’s resistance to azithromycin, erythromycin, clarithromycin, and penicillin were all very high, 64.9%, 65.9%, 63.3%, and 96.6%, respectively. The result for penicillin, in particular, which reached as high as 96.6%, may be related to its long-term use in hospitals as an antimicrobial. It is suggested that the resistance spectrum of *S. aureus* be further studied to find a new generation of antimicrobial drugs against these bacteria (see Table 2). The resistance rate of *S. aureus* to antimicrobial drugs showed the following: 214 cases of *S. aureus* were detected from 2022 Q1 to 2023 Q2, 119 cases of *S. aureus* were detected, and their resistance rate to antimicrobial drugs is shown in Table 3.

**Table 2:** The major *Staphylococcus aureus* resistance rate (%) during 2022Q1 to 2023Q2

	2022Q1	2022Q2	2022Q3	2022Q4	2023Q1	2023Q2
Azithromycin	71.4	52.9	60	63.2	82.6	59.1
Erythromycin	71.4	58.8	60	63.2	82.6	59.1
Clarithromycin	61.9	52.9	60	63.2	82.6	59.1
Penicillin	100	88.2	100	100	91.3	100

**Table 3:** The detailed resistance rate (%) of *Staphylococcus aureus* during 2022Q1 to 2023Q2

Antimicrobial	2022Q1 (*n* = 22)	2022Q2 (*n* = 17)	2022Q3 (*n* = 15)	2022Q4 (*n* = 20)	2023Q1 (*n* = 23)	2023Q2 (*n* = 22)
	Resistance rate (%)	Resistance rate (%)	Resistance rate (%)	Resistance rate (%)	Resistance rate (%)	Resistance rate (%)
Amikacin (loanword)	0	0	0	0	0	0
Azithromycin (macrolide antibiotic)	71.4	52.9	60	63.2	82.6	59.1
Benzazoline (antifungal agent)	36.4	5.9	13.3	45	43.5	22.7
Cotrimoxazole	13.6	11.8	0	0	0	0
Erythromycin (antibiotic)	71.4	58.8	60	63.2	82.6	59.1
Clarithromycin (antibiotic)	71.4	52.9	60	63.2	82.6	59.1
Clindamycin	61.9	29.4	53.3	47.4	65.2	13.6
Rifampicin (loanword)	0	0	0	0	0	0
Linezolid	0	0	0	0	0	0
Chloromycetin	0	0	0	5.3	0	0
Moxifloxacin	0	0	0	0	0	9.1
Penicillin (antibiotic)	100	88.2	100	100	91.3	100
Gentamycin (antibiotic)	0	0	0	5	8.7	9.1
Tetracycline	13.6	0	0	30	8.7	13.6
Tigecycline (antibiotic)	0	0	0	0	0	0
Ticoranin	0	0	0	10	0	0
Furantoin				0		
Norfloxacin	12.5	0	13.3	0	43.5	22.7
Vancomycin (antibiotic)	0	0	0	0	0	0
Levofloxacin	4.5	5.9	0	5	13	18.2

#### *Escherichia coli* resistance rate

*Escherichia coli* (*E. coli*) was identified by Gram-staining, an IMViC test, and a lactose fermentation test, followed by testing using the Vitek 2 automatic microorganism identifier to isolate bacterial strains and their matching identification cards. Among the *E. coli* bacteria detected, the resistance rate to ampicillin showed the highest percentage, which was stable at approximately 80%, suggesting that we should reduce the use of ampicillin against *E. coli*. Resistance to cotrimoxazole and ciprofloxacin was lower, while resistance to cefazolin showed a less stable state, suggesting that it can still be used to treat *E. coli* infections (see Table 4). The resistance rate of *E. coli* to antimicrobial drugs was as follows: 1,213 cases of *Enterobacterales* bacteria were detected from 2022 Q1 to 2023 Q2, among which *E. coli* was the most frequent (471 cases). The susceptibility rate of *E.coli* to antimicrobial drugs and drug-resistant bacteria is shown in Table 5.

**Table 4:** The maijor *Escherichia coli* resistance rate (%) during 2022Q1 to 2023Q2

	2022Q1	2022Q2	2022Q3	2022Q4	2023Q1	2023Q2
Ampicillin	85.7	89.6	30.4	88.2	83.5	82.4
Cotrimoxazole	69.8	70.8	73.9	69.7	61.5	52.9
Ciprofloxacin	54	60.4	65.2	48.7	50.5	46.1
Chloramphenicol	25	50	45.8	34.5	25	28.2
Cefazolin	75	57.1	66.7	62.1	37.5	53.8

**Table 5:** The detailed resistance rate (%) of *Escherichia coli* during 2022Q1 to 2023Q2

Antimicrobial	2022Q1 (*n* = 63)	2022Q2 (*n* = 48)	2022Q3 (*n* = 92)	2022Q4 (*n* = 75)	2023Q1 (*n* = 91)	2023Q2 (*n* = 102)
	Resistance rate (%)	Resistance rate (%)	Resistance rate (%)	Resistance rate (%)	Resistance rate (%)	Resistance rate (%)
Amikacin (loanword)	1.6	4.2	3.3	5.3	1.1	2
Ampicillin (loanword)	85.7	89.6	83.7	88.2	83.5	82.4
Ampicillin/sulbactam	39.7	29.2	30.4	26.3	23.1	13.7
Furantoin (antifungal agent)	0	0	2.9	2.1	1.5	4.8
Cotrimoxazole	69.8	70.8	73.9	69.7	61.5	52.9
Ciprofloxacin	54	60.4	65.2	48.7	50.5	46.1
Chloromycetin	25	50	45.8	34.5	25	28.2
Meropenem (loanword)	1.6	2.1	3.3	1.3	1.1	2.9
Minocycline (loanword)	4.8	4.2	9.8	5.3	2.2	4.9
Piperacillin/tazobactam	12.7	6.3	4.3	1.3	2.2	3.9
Gentamycin (antibiotic)	34.9	39.6	47.8	44.7	39.6	33.3
Ticarcillin/rodentic acid	28.6	14.6	14.1	18.4	6.6	7.8
Cefepime	19	20.8	30.4	31.6	26.4	25.5
Cefuroxime	41.3	41.7	51.1	57.9	44	41.2
Cefoperazone/sulbactam	7.9	6.3	4.3	3.9	6.6	6.9
Ceftriaxone	42.9	39.6	47.8	53.9	41.8	41.2
Ceftazidime (pharmacology)	27	29.2	27.2	32.9	23.1	31.4
Cefoxitin	6.3	8.3	8.7	13.2	4.4	9.8
Cefazolin (cephalexin), antifungal agent	75	57.1	66.7	62.1	37.5	53.8
Imipenem (tylenol)	1.6	2.1	3.3	0	1.1	2.9
Levofloxacin	33.3	47.9	60.9	42.1	47.3	43.1

#### ***Pseudomonas aeruginosa*resistance rate and the distribution of antibiotic resistance rate for*Pseudomonas aeruginosa*and*Acinetobacter baumannii***

*Pseudomonas aeruginosa* was identified by Gram-staining and oxidase and pyocyanin tests, followed by testing using the Vitek 2 automatic microorganism identifier to isolate bacterial strains and their matching identification cards. The resistance rates of *P. aeruginosa* to ciprofloxacin, gentamicin, ticarcillin and levofloxacin all showed very low percentages (below 15%) as shown in Table 6. This suggests that the resistance of *P. aeruginosa* in nosocomial settings is still within the acceptable range; nonetheless, attention should be focused on the resistance of this species as it is an important source of nosocomial infections. The resistance rate of *P. aeruginosa* to antimicrobial drugs was as follows: a total of 439 cases of non-fermenting bacteria were detected from 2022 Q1 to 2023 Q2, of which *P. aeruginosa* was detected in the highest number (288 cases) as shown in Table 7.

**Table 6:** The major *Pseudomonas aeruginosa* resistance rate (%) during 2022Q1 to 2023Q2

	2022Q1	2022Q2	2022Q3	2022Q4	2023Q1	2023Q2
Ciprofloxacin	6	9.7	17.2	4.7	9.1	8.9
Gentamicin	0	12.9	14.1	11.9	3.6	4.4
Ticarcillin	16.1	9.1	14.6	0	18.2	5.3
Levofloxacin	8	12.9	20.3	7.7	10.9	17.8

**Table 7:** The detailed resistance rate (%) of *Pseudomonas aeruginosa* during 2022Q1 to 2023Q2

Antimicrobial	2022Q1 (*n* = 50)	2022Q2 (*n* = 31)	2022Q3 (*n* = 64)	2022Q4 (*n* = 43)	2023Q1 (*n* = 55)	2023Q2 (*n* = 45)
	Resistance rate (%)	Resistance rate (%)	Resistance rate (%)	Resistance rate (%)	Resistance rate (%)	Resistance rate (%)
Amikacin (loanword)	4	3.26	1.6	0	7.3	6.7
Voltaren, a trade name for diclofenac sodium, an antifungal agent	14	6.5	4.7	11.6	7.3	6.7
Polymyxin B	0	0	4.2	3.8	3	0
Ciprofloxacin	6	9.7	17.2	4.7	9.1	8.9
Meropenem (loanword)	8	3.2	14.1	4.7	5.5	4.4
Piperacillin (loanword)	12.9	0	10.4	0	18.2	10.5
Piperacillin/tazobactam	12	3.2	7.8	7.7	10.9	2.2
Gentamycin (antibiotic)	0	12.9	14.1	11.9	3.6	4.4
Ticarcillin/baric acid	16.1	9.1	14.6	0	18.2	5.3
Cefepime	13	0	2.1	7.7	12.1	5.3
Cefoperazone/sulbactam	8	9.7	4.7	7.7	5.5	4.4
Ceftazidime (pharmacology)	8	6.5	12.5	4.8	14.5	17.8
Tobramycin (antibiotic)	0	3.2	10.9	11.9	5.5	4.4
Imipenem (tylenol)	13	0	14.6	0	9.1	10.5
Levofloxacin	8	12.9	20.3	7.7	10.9	17.8

**Fig. 3:**
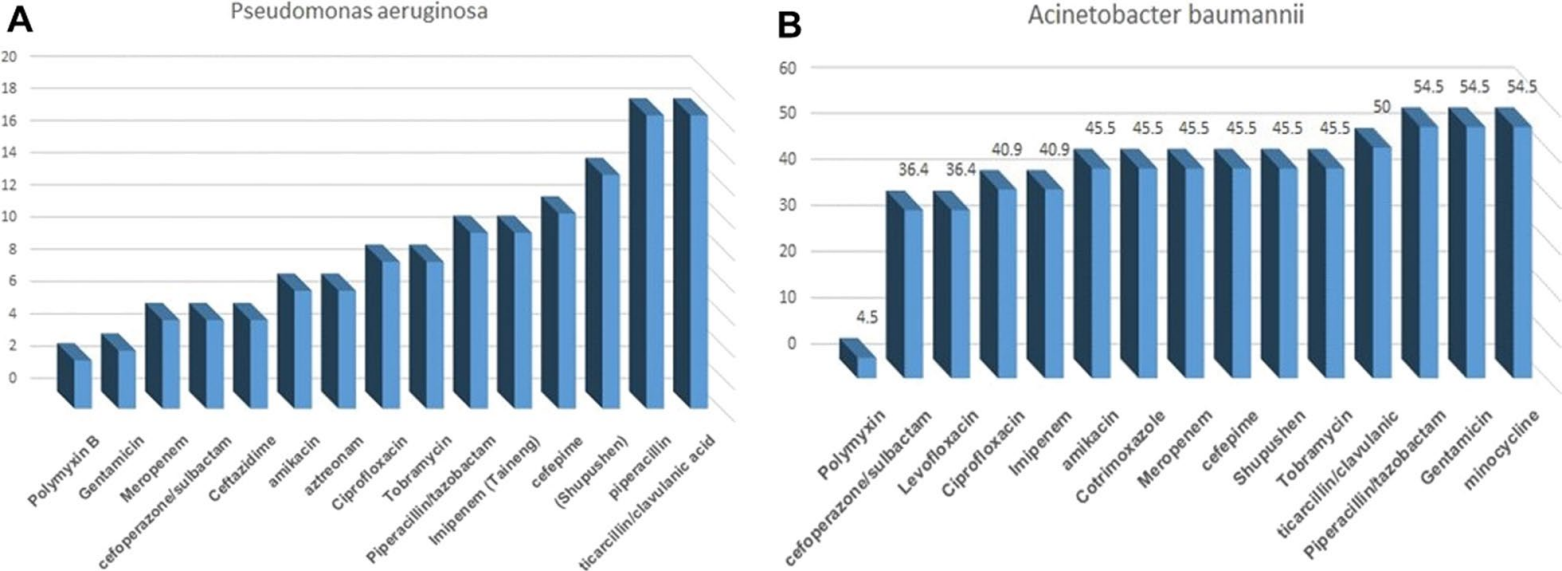
The distribution of resisted drug against *Pseudomonas aeruginosa* and *Acinetobacter baumannii*.  **A** The resistance rate of *Pseudomonas aeruginosa* against multiple drugs.  **B** The resistance rate of *Acinetobacter baumannii* against multiple drugs

Concerning the distribution of drug resistance, among the drugs we tested, *P. aeruginosa* showed lower drug resistance compared with *Acinetobacter baumannii* (*A. baumannii)*; this may have been because antibiotics were more carefully used against *P. aeruginosa* than against *A baumannii* in clinical settings. From the data, we observed a high resistance of *A. baumannii* to multiple drugs, which indicates the need for a stronger focus on the treatment of *A. baumannii* infections. The distribution of resisted drug against *Pseudomonas aeruginosa* and *Acinetobacter baumannii* is shown in Fig. 3. The patented drug used to treat *P. aeruginosa* and *A. baumannii* was polymyxin, which can be further investigated in clinical practice.

## Discussion

In this study, we analysed bacterial drug resistance in our hospital from 2022 Q1 to 2023 Q2. The most common Gram-positive bacteria detected in this study was *S. aureus*, followed by *E. faecalis*, *S. epidermidis*, and *S. haemolyticus*. No VR strains were detected, suggesting that vancomycin may be clinically indicated for the treatment of severe infections in MRSA and MR coagulase-negative *Staphylococcus*. The top three Gram-negative bacteria detected were *E. coli*, *K. pneumoniae*, and *P. aeruginosa*. Based on the monitored data, *Enterobacterales* bacteria still had a high sensitivity rate (> 90.0%) to carbapenem antimicrobial drugs; however, it remains a primary factor in causing severe patient infections. The data also showed that *P. aeruginosa* still has a high sensitivity rate (> 85.0%) to aminoglycoside antimicrobial drugs.

Multidrug-resistant bacteria were mainly found in respiratory specimens such as sputum (> 50%), which is consistent with reports in the literature [18, 19]. This may be related to the clinical practice of distributing samples, among which respiratory specimens accounted for about 50% of all specimens sent for testing. There is much controversy about the clinical value of bacterial cultures of sputum specimens, and the resistance rate of respiratory colonisers is generally higher than that of probable pathogens [20]; additionally, the number and proportion of MDR bacteria among the actual pathogens have yet to be confirmed. The second most common sample with bacteria detection is mid-stream urine and secretions, which is mainly related to the fact that clinical urinary tract infection pathogens are dominated by *E. coli* (ESBL-positive strains are numerous). Additionally, in secretion-infected specimens, the MRSA infection rate is relatively high, and contact with hospital-acquired MRSA carriers can increase the risk of MRSA colonisation [21], a factor that should be addressed in clinical settings. The spread of MRSA can be effectively controlled through a series of measures, such as strengthening MRSA screening and monitoring, and the timely isolation of patients with MRSA infections [22]. The isolation of bacterial strains from sterile bodily fluids such as blood has definite clinical significance; however, the proportion of MDR bacteria among these strains is much lower compared with the above-noted specimens, the reason for which is still unknown and requires further study and analysis.

The findings of our study are consistent with existing research that reported high prevalence and resistance rates for *S. aureus*, *E. coli*, *K. pneumoniae*, and *P. aeruginosa* in clinical settings [23–25]. These bacteria are known to cause various infections, such as skin and soft tissue infections, urinary tract infections, bloodstream infections, and respiratory infections, and pose a serious threat to public health [26]. The emergence and spread of MDR strains of these bacteria have reduced the treatment options and increased the morbidity and mortality of infected patients. Antimicrobial resistance surveillance studies are essential for providing timely and accurate information on the epidemiology and trends among resistant bacteria, thereby guiding the clinical diagnosis and treatment of infections, evaluating the effectiveness of infection prevention and control measures, and informing policymaking and resource allocation for antimicrobial stewardship programmes [27]. However, the quality and comprehensiveness of surveillance data can be affected by various factors, such as a lack of data on patient characteristics, clinical outcomes, and molecular mechanisms of resistance [28]. A comparative study on the changes of bacterial species and resistance rates to commonly used antibacterial drugs approximately 13 years ago showed that the resistance rates of the main Gram-positive cocci to commonly used antibacterial drugs have increased significantly over time [29]. Another study on the prevalence and drug resistance patterns of Gram-negative bacteria in a tertiary care hospital in India reported high resistance rates to commonly used antibiotics such as ceftriaxone, ciprofloxacin, amikacin, and piperacillin–tazobactam [30].

Isolation measures have been proposed to prevent ESBL infection, primarily active surveillance, the isolation of all patients infected with ESBL, contact prophylaxis for all colonised or infected patients, and the rational management of antimicrobial drugs [31]. A significant increase was found in the detection rate of ESBL bacilli, from 0.28 to 0.67‰ (*P* < 0.001) in admitted patients during this period, but the increase in the rate of nosocomial infection with ESBL was not high, suggesting that the infection control measures had a role in controlling the nosocomial transmission. Hassoun et al. [32] achieved a significant improvement in the detection rate of ESBL bacilli from 0.28 to 0.67‰ (*P* < 0.001) among patients over 2 years through a series of MRSA control measures. The incidence of MRSA hospital-acquired infections among inpatients decreased from 0.7/1000 hospital days in the first quarter of 2007 to 0.29/1000 hospital days in the fourth quarter of 2008 (*P* = 0.05), showing a 59% reduction in the transmission of MRSA hospital-acquired infections. The existing literature and relevant national regulations and guidelines indicate that hospital infections can be effectively prevented and controlled through, for example, the adoption of targeted surveillance, strict hand hygiene measures, disinfection and isolation, the education of medical staff, and effective supervision. However, to achieve good results, not only one department or one method can be implemented; rather, the cooperation of all hospital departments, collaboration among the various personnel, and the application of multiple methods should be adopted [33].

This study has some limitations, such as a lack of data on patient characteristics, clinical outcomes, and molecular mechanisms of resistance. Future studies should address these gaps and improve the quality and comprehensiveness of surveillance data.

## Conclusion

In summary, this study provides valuable insights into bacterial drug resistance surveillance in a hospital in China from 2022 Q1 to 2023 Q2. The study identified the common bacterial genera and their resistance patterns in different specimen types and suggests the need for the more rational use of antimicrobials and enhanced infection prevention and control measures in the hospital. The study also demonstrates the usefulness of surveillance data for informing clinical practice and policymaking and calls for further research to improve the surveillance system.

## Data Availability

No datasets were generated or analysed during the current study.
